# Thermal biology of *Hypogeococcus pungens* (Hemiptera: Pseudococcidae) explains its variable performance as a classical biological control agent for *Harrisia martinii* (Cactaceae) in Australia

**DOI:** 10.1093/ee/nvaf026

**Published:** 2025-04-12

**Authors:** Angela E Ezeh, Myron P Zalucki, Michael D Day, Tamara Taylor, Michael J Furlong

**Affiliations:** School of the Environment, The University of Queensland, St Lucia, Queensland, Australia; School of the Environment, The University of Queensland, St Lucia, Queensland, Australia; Department of Agriculture and Fisheries, Ecosciences Precinct, Brisbane, Queensland, Australia; Department of Agriculture and Fisheries, Ecosciences Precinct, Brisbane, Queensland, Australia; School of the Environment, The University of Queensland, St Lucia, Queensland, Australia

**Keywords:** climate, climex model, insect development, temperature

## Abstract

The mealybug, *Hypogeococcus pungens* Granara de Willink (Hemiptera: Pseudococcidae), was released in Australia as a biological control agent for *Harrisia martinii* (Labour.) Britton (Cactaceae) in 1975. Although the mealybug successfully established in all released locations, its impact has been variable among regions, possibly as a result of climatic differences. Life-history traits (settling time, survival, development time, female reproduction, adult longevity) were compared at 6 constant temperatures (15 to 40 °C) in the laboratory. The mealybug settled on *H. martinii* at all temperatures tested, but at 15 °C and 40 °C, insects failed to develop and died. Temperature affected female size, fecundity, and integrated performance, all of which were highest at 25 °C. A linear model that fitted temperature to development time indicated a lower developmental threshold of 14.5 °C for both male and female mealybugs. CLIMEX models were developed for the mealybug and its host, *H. martinii,* and used to investigate the suitability of different regions of Australia, where *H. martinii* occurs for *Hy. pungens.* The *Hy. pungens* CLIMEX model suggests that cold stress limits mealybug growth in southern Queensland and that mealybug performance will vary between regions based on local temperatures. Locations with extreme low winter and extreme high summer temperatures are likely to have the most constrained populations. This may account for the observed differences in the effectiveness of *Hy. pungens* as a biological control agent at locations within the established range of *H. martini* in Australia.

## Introduction

The columnar cactus *Harrisia martinii* (Labour.) Britton (Cactaceae) originates from the Chaco region of Argentina. Originally introduced into Australia in 1900 as an ornamental plant (Mann 1967), *H. martinii* was recorded invading Australian rangelands in the Collinsville district of central Queensland in 1935 (Mann 1967). The species is now classified as a restricted invasive weed in Queensland and New South Wales (Biosecurity Queensland 2017, DPI New South Wales 2020) because of its ecological and economic impacts on pastoral agriculture, cropping, and biodiversity (Mann 1967, Houston and Elder 2019).

In 1975, a cactus mealybug, previously identified as *Hypogeococcus festerianus* (Lizer y Trelles) (Hemiptera: Pseudococcidae), was introduced into Australia from the Chaco region of Argentina for the biological control of *H. martinii* (McFadyen 1979). This mealybug was later re-described as *Hypogeococcus pungens* Granara de Willink (Hemiptera: Pseudococcidae) (Williams and Willink 1992), a species complex comprising at least 5 genetically distinct populations that are adapted to different plant hosts (Poveda-Martínez et al. 2020). The insects introduced into Australia originated from Cactaceae native to Argentina (McFadyen 2012) and belong to the *Hy. pungens* species complex (Ezeh et al. 2023). The immature stages of the Australian *Hy. pungens* consist of 3 nymph stages in females and 4 stages in males. The potential fecundity of females is 80 to 100 eggs, with a pre-oviposition period of 20 d and an oviposition period of up to 35 d. Females lay 2 to 4 eggs per day, with egg hatching occurring within 20 min (McFadyen 1979). Nymphs settle at spine bases, in cracks between stem ribs, and on buds of *H. martinii*. Once settled, female nymphs remain in this position throughout their lives but male nymphs move to more exposed parts of the plant (spines and stem surface) after the second instar stage where they pupate within a cylindrical cottony cocoon. Adult males are winged and highly mobile to facilitate mating with females (McFadyen 1979).

*Hypogeococcus pungens* feeds on the tips of stems and flower buds of *H. martinii*, damaging the growing tissue, preventing flowering and seed production, and arresting further growth (McFadyen 1986, 2012). In response to mealybug attack, *H. martinii* uses the energy reserves in its tuberous root to produce new growth (Tomley and McFadyen 1985) but plants can die when re-growth cannot be sustained as a result of the depletion of resources (McFadyen 2012). A post-release evaluation of *Hy. pungens* in 1984 compared the impact of the mealybug on *H. martinii* at original release sites at Collinsville in central Queensland and at Goondiwindi in southern Queensland, using changes in the percentage of *H. martinii* cover at the sites to estimate agent efficacy (Tomley and McFadyen 1985). Plant cover was reduced from 50% to less than 0.5% at sites in central Queensland, while an increase in plant cover from 15% to 84% was reported at sites in southern Queensland (Tomley and McFadyen 1985, McFadyen 1986). The reasons for this apparent geographically variable success were not determined in previous studies.

Interactions between multiple biotic and abiotic factors have been suggested to impact the performance of some biological control agents (Byrne et al. 2002, McClay and Hughes 2007, Manrique et al. 2008, Triapitsyn et al. 2018), leading to variable outcomes of introductions. Among these, temperature is often regarded as a key abiotic factor due to its direct effects on insect population dynamics (Coulson and Bale 1992, Bale et al. 2002) and plant-insect interactions (Bale et al. 2002). The impacts of temperature on insects and their responses to such changes vary between species. Generally, temperature affects insect life cycles and phenology (Zachariassen 1985, Robinet and Roques 2010) and studies have shown that temperature can play a critical role in the establishment, proliferation, and subsequently, performance of biological control agents (Byrne et al. 2002, Zalucki and van Klinken 2006, Manrique et al. 2008, Avila and Charles 2018). To increase the likelihood of success of biological control, agents should be sourced from an area with similar climatic conditions to the area in which they are to be released. This helps to reduce the likelihood of post-release temperature shocks and increases the chance of establishment and subsequent effectiveness of the agent (Robertson et al. 2008, Olfert et al. 2016). For example, the gall wasp, *Trichilogaster acacielongifoliae,* Froggatt (Pteromalidae) imported from Australia for the biological control of *Acacia longifolia* (Andrews) Willd. (Fabaceae) in South Africa, established and performed better in areas with a climate similar to that in its native range (Dennill and Gordon 1990).

Studies on the impact of temperature on the biotic potential and performance of biological control agents are often conducted post-release, especially when the agent fails to establish and/or perform optimally in the introduced range (Harms et al. 2021, Cowie et al. 2023). In classical biological control, some predictive ecoclimatic models have been utilized to visualize the potential distribution and population dynamics of the introduced (or potential) agent in the target environment (Zalucki and Klinken 2006, Stephens et al. 2007, Hemerik and van Nes 2016, Furlong and Zalucki 2017, Avila and Charles 2018). CLIMEX is a modeling package that can be used to predict the geographical distribution, seasonal phenology, and relative abundance of a species based on its biology and historical or predicted climatic data (Sutherst and Maywald 1985).

Currently, there are no studies on the potential distribution of either *H. martinii* or *Hy. pungens* in Australia. As part of efforts to understand the variable performance of *Hy. pungens* as a biological control agent of *Harrisia martinii* in Australia, the effect of temperature on the biotic potential of the mealybug on *H. martini* was assessed using the recognized indices of development time, fecundity, and longevity (Southwood and Henderson 2000). Laboratory experiments were conducted to determine the effects of a range of constant temperatures (15, 20, 25, 30, 35, and 40 °C) on settling, development time, survival, fecundity, and longevity of *Hy. pungens*. The data were then used to estimate the integrated performance, thermal constant, and lower developmental threshold for the species and to predict the performance of the mealybug at different locations in Australia compared with its native range. The distributions of *H. martinii* and *Hy. pungens* in their native ranges were used to parameterize separate CLIMEX models (Sutherst and Maywald 1985, Kriticos et al. 2015) for the weed and its biological control agent. The models were then used to examine the suitability of the Australian climate for these organisms and to investigate the different stress factors that might act on them in different parts of their Australian distributions.

## Materials and Methods

### Maintenance of Plants and Insects

*Harrisia martinii* plants used to maintain insect cultures and those used in all experiments were collected in Ipswich, southeast Queensland (27.3321°S, 152.4762°E) in December 2019. Eighty (80) young, insect-free *H. martinii* stem cuttings (20 cm long), collected individually from different *H. martinii* clumps were potted in an organic potting medium in 140 mm diameter plant pots and maintained at ambient temperature (26 ± 5 °C) and light conditions (12:12 h, L:D) in a glasshouse at The University of Queensland, St Lucia, Queensland. *Hypogeococcus pungens* collected from *H. martinii* in Goondiwindi, southern Queensland (28.4925°S, 150.2501°E), was established on potted *H. martinii* plants and maintained under ambient conditions (24 ± 2 °C; 12:12 h, L:D; RH 40 to 55%) in the laboratory for 9 mo before the experiment.

### Effects of Temperature on Mealybug Life-history Traits

To determine the effects of temperature on settling, nymph development, and other traits, newly emerged (0 to 12 h old) first-instar nymphs (*n* = 15 per plant) were placed singly onto *H. martinii* plants grown on an organic potting medium in 140 mm diameter pot. Fifteen first-instars were placed on each of 48 plants. Then groups of 8 infested plants were randomly chosen and placed in a fine mesh (44 × 32 mesh) cage (W100 × D100 × H100) and each cage was transferred to one of 6 different constant temperatures (15, 20, 25, 30, 35, and 40 °C; 12:12 h, L:D; RH 50-70%) in controlled environment chambers for insect rearing. Temperature data loggers (Lascar EL-21CFR-1-LCD) were used to monitor temperatures inside the chambers. The experiment was conducted between October 2020and May 2021.

#### Settling Time and Survival

Settling time was recorded as the time from the introduction of the mealybug nymphs to the host plants to the onset of the whitish cottony wax production, which indicates successful settling at a microsite (McFadyen 1979). Using a fine-tip permanent marker, a mark was made very close to each settled nymph for easy recognition. The nymphs were observed daily until all had settled. Survival was recorded at two-time points, at settling (the proportion of introduced nymphs that settled), and to adulthood (the proportion of settled nymphs that completed development to adulthood).

#### Development Time, Lower Development Threshold, and Thermal Constant

A further observation was made daily on each marked settled nymph and other life history traits were recorded. The time taken to complete development to the second instar for each settled nymph was recorded as the time from the introduction of the neonate nymphs onto the plants to the appearance of the first exuvium. The development times for other instars were recorded as the time from the appearance of the previous exuvium to the appearance of the next exuvium or adult emergence (for males) and third molt for females. The cast-off exoskeleton was carefully removed with a fine paintbrush following molts to successive instars. The overall development time was the total development time from the first instar to the eclosion of adults.

The lower development threshold (*t*_o_) is the temperature below which no development occurs, while the thermal constant (*K*) is the number of degree-days required by an ectotherm to complete development to a particular stage or its complete life cycle. These thermal parameters were estimated for each immature stage of *Hy. pungens* separately and for the total period of development through the immature stages to adulthood using the linear model of Campbell et al. (1974). In this model, the inverse of the development time (= rate of development) (*R*), is plotted against the rearing temperature (*T*). The relationship between the development rate and the temperature can be expressed as *R(T) = a + bT*, where *a* is the intercept and *b* is the regression coefficient or slope. The lower development threshold is calculated as the ratio of the intercept and regression coefficient (*a/b*), while the thermal constant is calculated as the inverse of the regression coefficient (*1/b*). Non-linear models were not considered appropriate as there were too few temperatures around the optimum temperature and the threshold.

#### Female Reproduction

Following adult eclosion, females were observed daily for signs of emerging nymphs, as mating occurs immediately after the emergence of males and eggs hatch rapidly after oviposition (≤20 min) (McFadyen 1979).

To assess female reproductive potential, the following traits were investigated:

i) *Oviposition periods and realized fecundity*: 10 mated females from each temperature regime were randomly selected for observation. Using fine-tipped permanent marker pens of different colors, a mark was made close to each selected female. One or two females were marked per host plant, and all other females on the test plant were removed on emergence of the first nymph. For each marked female, the pre-oviposition and the oviposition periods were recorded. The realized fecundity was measured by observing each female daily from initial eclosion to death and recording and removing all the nymphs that she produced.

ii) *Female size and potential fecundity*: At emergence of the first nymph on a plant, ten mated females (yet to produce nymphs) were randomly selected from each temperature regime, removed from the test plants, and the length (*h*) and width (=diameter, D) of each was measured using a binocular stereo microscope (Meiji EMZ 65063, Meiji Techno co. Ltd, Tokyo, Japan). Assuming that the females approximated a cylinder in shape, the volume (V) of each female was then calculated according to:

V = *π(D/2)*^*2*^*h.*

Each measured female was then placed in 70% ethanol and later dissected using an entomological pin, under the binocular stereo microscope. The number of eggs in each female was recorded as an estimate of potential fecundity.

#### Adult Lifespan

At each test temperature, the lifespan of 10 adult males and 10 adult females was determined by daily observation of individuals from the point of eclosion until they died. For the female lifespan, the 10 marked adult females used for the realized fecundity study were observed until death. Dead females were easily recognized by a change in their body color from pink/light brown to black upon death. Since adult males are highly mobile, it was difficult to mark and trace them individually. Therefore, at adult eclosion, they were isolated and each adult male was placed into a Petri dish (9 cm diameter) that was covered with a fine mesh net (for aeration) and held in position with a rubber band. Death of males was confirmed by lack of movement when the body was touched with a fine paintbrush.

### Integrated Performance of *Hy. pungens*

The *Hy. pungens* fitness or performance at each temperature was calculated and compared using the integrated performance model. Such models integrate an organism’s life-history traits, including sex ratio, development, and reproductive rates, to estimate its potential population growth at a given temperature (Angilleta 2009) and have been used to determine the performance of biological control agents (for example see, Mathenge et al. 2009, Wang et al. 2020, 2022). In this study, the performance (P) of *Hy. pungens* at each test temperature was estimated by:


$$\begin{array}{l} P=\frac{F*S}{DL} \end{array}$$

where DL = development time (days) (first instar to the start of female reproduction), F = realized fecundity, adjusted by the sex ratio of offspring (realized fecundity multiplied by 0.6), S = mean survival (%).

### Comparisons of Climate Data and Critical Temperature Parameters of *Hy. pungens* at Sites of Origin and Sites of Introduction

Long-term average monthly minimum, maximum, and mean temperature data were obtained for Goondiwindi, Collinsville (Lat. 20.5531°S, Long. 147.8443°E) in Queensland, Australia, and Chaco (Lat. 27.4257°S, Long. 59.0244°W), north-east Argentina (Australian Meteorology 2021, Global Historical Weather and Climate 2022). Data for each location were plotted separately and for each place, (i) the area (°C × month) under the mean monthly temperature curve that fell below the mealybug’s lower temperature threshold, (ii) the area under the mean minimum monthly temperature curve that fell below the mealybug’s lower temperature threshold, and (iii) the sum of the area of the mean monthly temperature curve that fell above and below the optimal temperature for the mealybug’s performance, were calculated and compared.

### Predicting the Performance of *Hypogeococcus pungens* and *Harrisia martinii* in Australia Using CLIMEX

In CLIMEX modeling, a species’ response to climate is represented by the growth index (GI) and various stress indices. The GI is a product of temperature and moisture indices and indicates the potential growth rate of a species in a location (Sutherst and Maywald 1985, Sutherst 2004). The temperature index (TI) indicates a species’ response to daily temperature changes and is a linear function defined by 4 critical temperatures: the limiting low temperature (DV0) is the temperature threshold below which no population growth occurs, the lower (DV1) and the upper (DV2) optimal temperatures define the temperature range where population growth is maximal (=1), and the high-temperature threshold (DV3) is the temperature above which no growth occurs. The moisture index (MI) indicates a species’ response to soil moisture and is defined by 4 parameters: the limiting low soil moisture (SM0) represents soil moisture below a critical threshold where no growth occurs, the lower (SM1) and upper (SM2) optimal soil moisture define the soil moisture levels where growth is maximal, while above the limiting high soil moisture (SM3) growth is zero.

Stress indices (cold, hot, wet, and dry indices) describe the effects of unfavorable seasons on species survival (Sutherst and Maywald 1985, Sutherst 2004). The stress indices have 2 parameters: a threshold parameter which indicates when stress begins to accumulate and a rate parameter which determines the rate at which stress is accumulated. The cold stress (CS) consists of the cold stress threshold (TTCS) and cold stress temperature rate (THCS). Heat stress (HS) consists of the heat stress temperature threshold (TTHS) and heat stress temperature rate (THHS). Dry stress (DS) consists of the dry stress threshold (SMDS) and dry stress rate (HDS) while wet stress (WS) consists of the wet stress threshold (SMWS) and wet stress rate (HWS).

The combination of growth and stress indices determines the Ecoclimatic Index (EI), which is scaled from 0 to 100 and describes the climatic suitability of a location for a species (EI = 100 indicates optimal climate for species persistence, while EI = 0 indicates that a species cannot persist at a location) (Sutherst and Maywald 1985, Kriticos et al. 2015).

Based on the known distribution records of *Hy. pungens* and *H. martinii* in South America, and laboratory data on *Hy. pungens* generated in this study, a CLIMEX model (Sutherst and Maywald 1985, Kriticos et al. 2015) was developed to describe the potential distribution of the mealybug and its host plant in Australia. The CLIMEX parameter values (Table 1) were iteratively fitted to the known distributions of both species in South America and the stress-related parameter values were adjusted to fit these distributions while being mindful that they need to be biologically reasonable (Kriticos et al. 2015). The model parameters were then used to predict the distributions of *Hy. pungens* and *H. martinii* in Australia and to investigate their seasonal phenologies at locations of interest.

**Table 1.** CLIMEX parameter values for Hypogeococcus pungens and Harrisia martinii^a^

Parameters		*Hy. pungens*	*H.martinii*
**Moisture parameters**			
Limiting low soil moisture	SM0	0.1	0.1
Lower optimal soil moisture	SM1	0.2	0.2
Upper optimal soil moisture	SM2	1.5	0.8
Limiting high soil moisture	SM3	2	1
**Temperature parameters**			
Limiting low temperature	DV0	15	10
Lower optimal temperature	DV1	25	20
Upper optimal temperature	DV2	28	30
Limiting high temperature	DV3	40	40
**Stress parameters**			
**Cold stress**			
Cold stress threshold	TTCS	12	10
Cold stress temperature rate	THCS	0	-0.001
Cold stress Degree-day threshold	DTCS	20	Not used
Cold stress Degree-day rate	DHCS	−0.00025	Not used
**Heat stress**			
Heat stress temperature threshold	TTHS	40	40
Heat stress temperature rate	THHS	0.001	0.001
**Dry stress**			
Dry stress threshold	SMDS	0.01	0.1
Dry stress rate	HDS	−0.1	−0.025
**Wet stress**			
Wet stress threshold	SMWS	2	1
Wet stress rate	HWS	0.02	0.02

^a^See CLIMEX User Manual (Kriticos et al. 2015) for details of equations and further description of parameters.

### Data Analysis

Statistical analyses of the data were conducted in GraphPad Prism (version 8.0.1). The normality of the distribution of dependent variables was tested using the Shapiro–Wilks test before conducting all statistical analyses. When data were not normally distributed (settling time of nymphs, survival from settling to adult, oviposition period, fecundity, and integrated performance), they were analyzed using the non-parametric Kruskal–Wallis test, and posthoc analyses were conducted using Dunn’s multiple comparisons test. The effect of temperature on survival at settling and oviposition period was tested using a one-way ANOVA and posthoc analyses were conducted using Tukey’s multiple comparison test. The relationship between female size and potential fecundity was tested using Spearman’s correlation. The relationship between development rate and temperature was tested using linear regression, while adult male and female survivorship curves were compared using the log-rank (Mantel-Cox) test.

## Results

### Effects of Temperature on Mealybug Life-history Traits

#### Settling Time and Survival

**Fig. 1.**
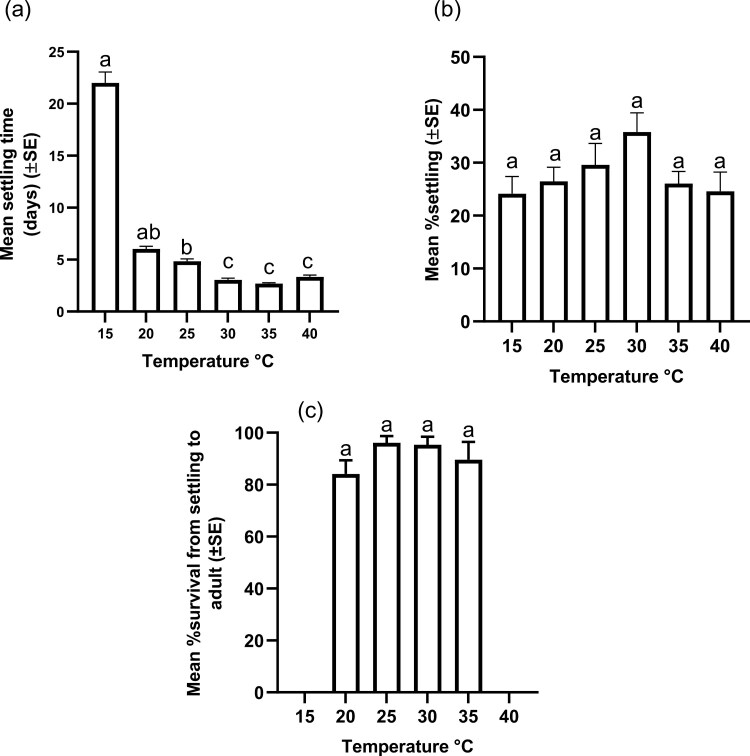
The mean (±SE) settling time (days) of *Hy. pungens* on *H. martinii*. The number of days to settle at different constant temperatures was significantly affected by temperature (Kruskal–Wallis test, *H* (6) = 125.83, *P* < 0.0001); columns marked with different letters are significantly different (Dunn’s multiple comparisons test, *P < *0.05); (b) The mean (±SE) percentage of *Hy. pungens* settling was not significantly affected by temperature (*F*_5,42_ = 1.76, *P* = 0.142); (c) The mean (±SE) percentage survival of *Hy. pungens* from settling to adult. No insects completed development at 15°C or 40°C; within the temperature range of 20 to 35 °C survival was not affected by temperature (Kruskal–Wallis test, *H* (4) = 4.309, *P* = 0.230).

*Hypogeococcus pungens* settled on *H. martinii* at all test temperatures. However, temperature significantly affected the time to settling (Kruskal-Wallis test, *H* (6) = 126.3, *P *< 0.0001), with nymphs taking longer to settle at lower temperatures (Fig. 1a).

Survival at settling (% settling) was ≤36% at all temperatures and was not affected by temperature (*F*_5,42_ = 1.76, *P* = 0.142) (Fig. 1b). None of the nymphs that settled at 15 or 40 °C survived to adulthood (Fig. 1c). At all other temperatures, >80% of insects that settled completed development to adulthood, and within this temperature range (20 to 35°C), survival was not affected by temperature (Kruskal–Wallis test, *H* (4) = 4.309, *P* = 0.230).

#### Development Time, Lower Development Threshold, and Thermal Constant

**Fig. 2.**
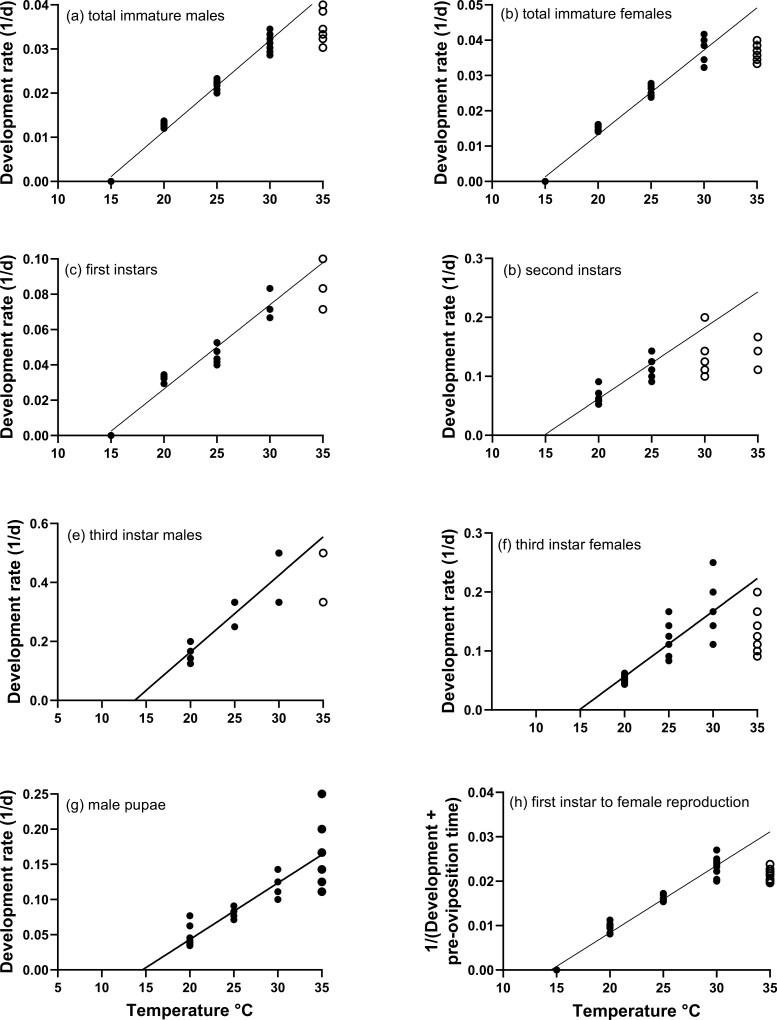
The linear relationships between temperature and developmental rate (1/days) for each life stage of *Hypogeococcus pungens;* (a) total immature male development; (b) total immature female development; (c) first instar nymph development; (d) second instar nymph development; (e) third instar male nymph development; (f) third instar female nymph development; (g) male pupa development; (h) development from first instar to start of female reproduction. (Note open dots in the graph represent data that are not part of the linear regression as they are not linearly related to temperature.)

*Hypogeococcus pungens* completed development from neonate nymph to adult at 20, 25, 30, and 35 °C and the times for both sexes to complete development decreased with increasing temperature (Fig. 2). The lower development threshold (*t*_o_) for neonates to develop to second instars and for male and female insects to complete development to adults was estimated to be 14.5 °C (Table 2). The thermal constant (K) for *Hy. pungens* to complete development was estimated at 486- and 418-degree days for males and females respectively (Table 2).

**Table 2.** Linear regression parameter estimates for development threshold temperature to (^o^C) and development duration K (degree days), describing the relationship between temperature and development rate (1/days) of *Hypogeococcus pungens*

Stages	Intercept	Slope	t_o_	K	*r* ^2^	*P*-value
First nymph	−0.06907	0.00477	14.5	209.7	0.9495	< 0.0001
Second nymph	−0.17930	0.01207	14.9	82.9	0.8438	< 0.0001
Third nymph female	−0.16480	0.01107	14.9	169.5	0.7604	< 0.0001
Third nymph male	−0.35650	0.02603	13.7	38.4	0.7850	< 0.0001
Male Pupa	−0.11730	0.00803	14.6	124.6	0.7424	< 0.0001
First nymph- adult male	−0.02977	0.00206	14.5	486.2	0.9842	< 0.0001
First nymph- adult female	−0.03460	0.00239	14.5	417.9	0.9733	< 0.0001
First nymph- reproduction	−0.02189	0.00151	14.5	660.4	0.9705	< 0.0001

#### Female Reproduction

**Fig. 3.**
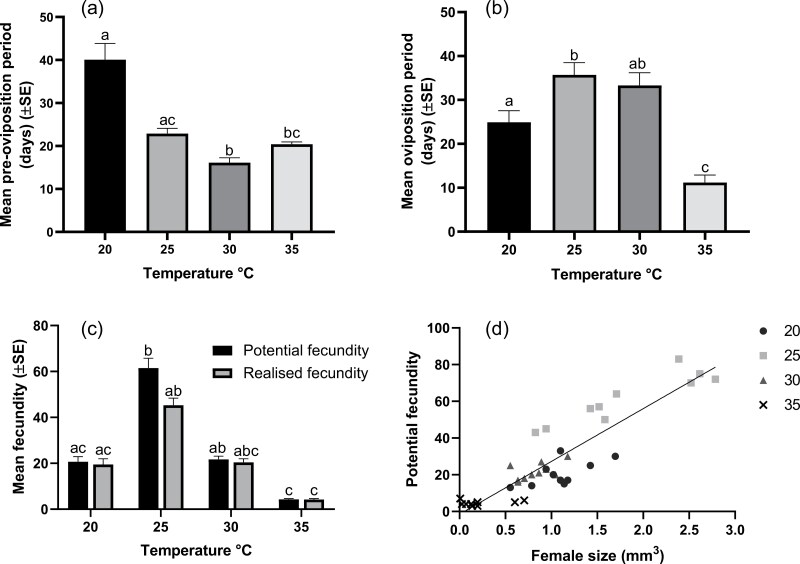
(a) The mean (±SE) preoviposition period of females of *Hypogeococcus pungens*. There was a significant difference between the preoviposition periods (Kruskal–Wallis test, *H* (4) *=* 29.047*, P < *0.001) at different temperatures. Means labeled with different letters are significantly different from each other (Dunn’s multiple comparison test, *P *< 0.05); (b) The mean (±SE) oviposition period was significantly affected by temperature (*F*_3,36_ = 26.18, *P *< 0.001); means labeled with different letters are significantly different from each other (Tukey’s multiple comparisons test, *P *< 0.05); (c) The mean (±SE) potential and realized fecundities of female *Hy. pungens* were significantly affected by temperature (Kruskal–Wallis test, *H* (8) = 67.043, *P* < 0.001); means labeled with different letters are significantly different from each other (Dunn’s multiple comparisons test, *P < 0.05*) and, (d) The correlation between female size and potential fecundity (*r* (38) = 0.86, *P* < 0.001).

Temperature significantly affected the pre-oviposition period (Kruskal–Wallis test, *H* (4) = 29.047, *P* < 0.001) (Fig. 3a), and oviposition period (*F*_3,36_ = 26.18, *P < *0.001) (Fig. 3b). The pre-oviposition period was longest at 20 °C (40 ± 12 d) and shortest at 30 °C (16 ± 4 d). The oviposition period was longest at 25 °C, with a mean of 36 ± 9 d. Temperature significantly affected both potential fecundity and realized fecundity (Kruskal–Wallis test, *H* (8) = 67.043, *P* < 0.001) (Fig. 3c), both of which were highest at 25 °C. There was a positive relationship between female size and potential fecundity (*r* (38) = 0.86, *P* < 0.001) across all rearing temperatures (Fig. 3d)

#### Adult Lifespan

**Fig. 4.**
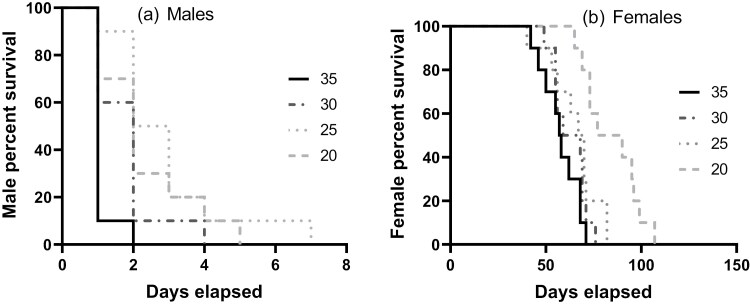
Survivorship curves for (a) adult male and (b) adult female Hypogeococcus pungens at 4 constant temperatures. Adult male survival (Log-rank test for trend, X^2^ = 6.246, df = 1, *P* = 0.0124) and adult female survival (Log-rank test for trend, X^2^ = 19.83, df = 3, *P* = 0.002) were each significantly affected by rearing temperature.

The mean lifespans of adult male and female insects increased with decreasing temperature. However, the male lifespan declined at temperatures below 25 °C. Adult males had a shorter lifespan than females, with significant differences across test temperatures (Log-rank test for trend, *X*^*2*^*=* 6.246*, df =* 1*, P = *0.0124). Males lived longer at 25 °C (mean lifespan = 3 ± 1.7 d) and lived for the shortest periods at 35 °C (1 ± 0.3 d) (Fig. 4a). The longest female mean lifespan was recorded at 20 °C (84 ± 15 d) and the shortest female lifespan was recorded at 35 °C (57 ± 10 d) (Log-rank test for trend, *X*^*2*^ = 19.83, df = 3, *P* = 0.002) (Fig. 4b).

### Integrated Performance of *Hy. pungens*

**Fig. 5.**
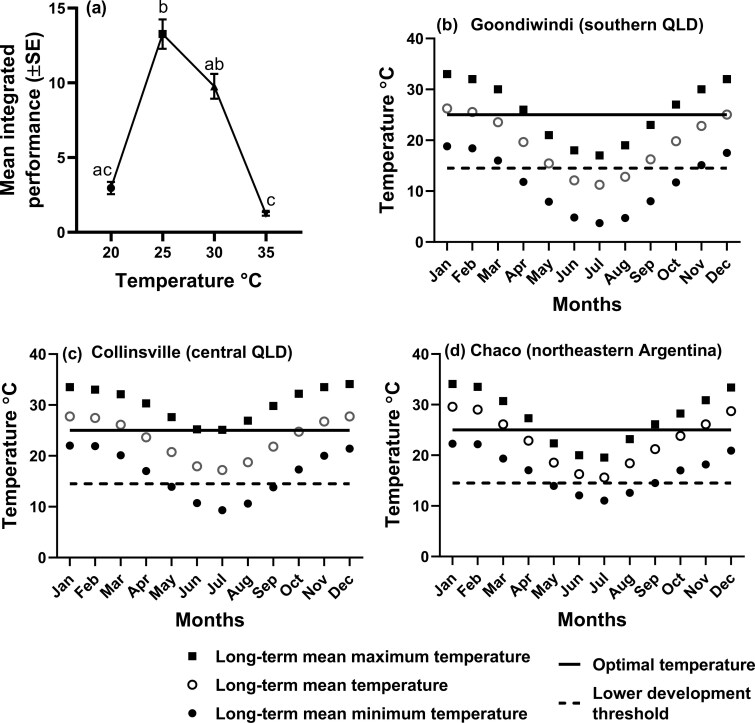
(a) The mean (±SE) integrated performance of *Hypogeococcus pungens* at different constant temperatures (Kruskal–Wallis test, *H* (4) = 35.022, *P* < 0.001). Means labeled with different letters are significantly different from each other (Dunn’s multiple comparison test, *P < *0.05); (b–d) Long-term average monthly minimum, monthly maximum, and monthly mean temperatures at Goondiwindi, Collinsville, and Chaco respectively.

The integrated performance of *Hy. pungens* varied significantly with temperature (Kruskal–Wallis test, *H* (4) = 34.08, *P < *0.0001), with no significant difference between performance at 20 and 30 °C. Integrated performance was greatest at 25 °C, while the lowest performance was recorded at 35 and 20 °C (Fig. 5a).

### Comparisons of Climate Data and Critical Temperature Parameters of *Hy. pungens* in Region of Origin and at Sites of Introduction

At Goondiwindi, the area of the long-term average mean monthly temperature curve that was below *t*_o_ for *Hy. pungens* was 7.35 degree-months (°C × month) (Fig. 5b and Table 3), while the long-term average mean monthly temperature curves at Collinsville and Chacos were never below this critical temperature (Fig. 5c, d and Table 3). Similarly, the area of the long-term average minimum monthly temperature curve at Goondiwindi below this critical temperature was 48.9 degree-months (Fig. 5b and Table 3), an area 3.4-fold and 5.9-fold greater than the areas under the corresponding curves at Collinsville and Chacos, respectively (Fig. 5c,d and Table 3). At Goondiwindi, the area of the long-term average mean monthly temperature that was above or below the optimal temperature for *Hy. pungens* performance was 73.2 degree-months, an area 1.8-fold and 1.4-fold greater than the areas above or below the corresponding curves at Collinsville and Chacos, respectively (Fig. 5c,d and Table 3).

**Table 3.** Relationships between long-term average monthly mean temperatures and long-term average monthly minimum temperatures and the lower development temperature threshold (t_o_) and optimal temperature (opt) at Goondiwindi, Collinsville, and Chacos.

Location	Area^a^ of average mean temperature curve below t_o_	Area^a^ of average minimum temperature curve below t_o_	Area^a^ of average mean temperature curve above/ below t_opt_
Goondiwindi	7.4	48.9	73.2
Collinsville	0	14.2	40.9
Chacos	0	8.3	52.7

^a^Units= °C × month.

### Predicting the Performance of *Hypogeococcus pungens* and *Harrisia martinii* in Australia Using the CLIMEX Compare Location Model

**Fig. 6.**
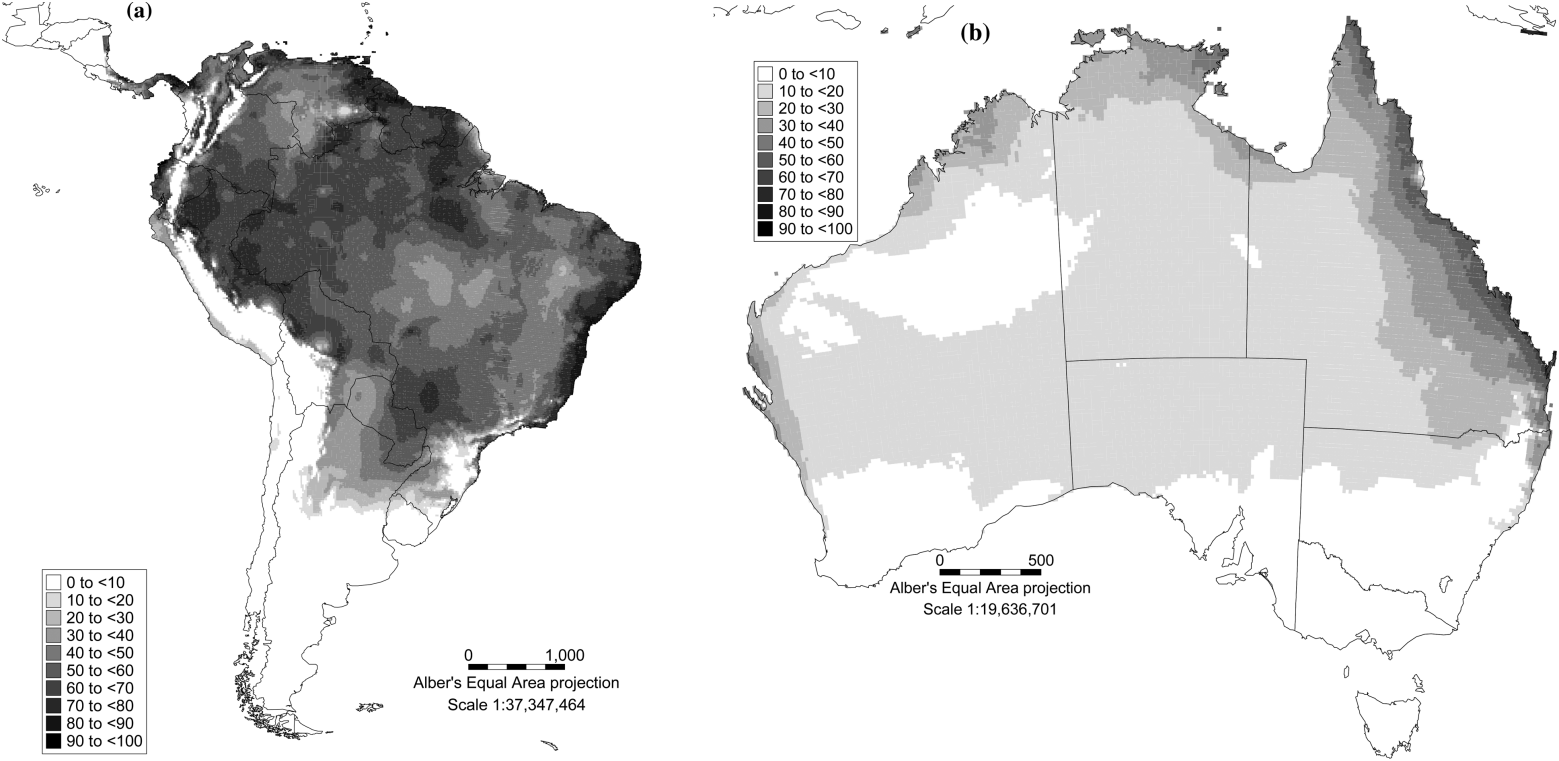
Ecoclimatic suitability (EI) for the; (a) mealybug *Hypogeococcus pungens* in South America; (b) mealybug *Hypogeococcus pungens* in Australia.

**Fig. 7.**
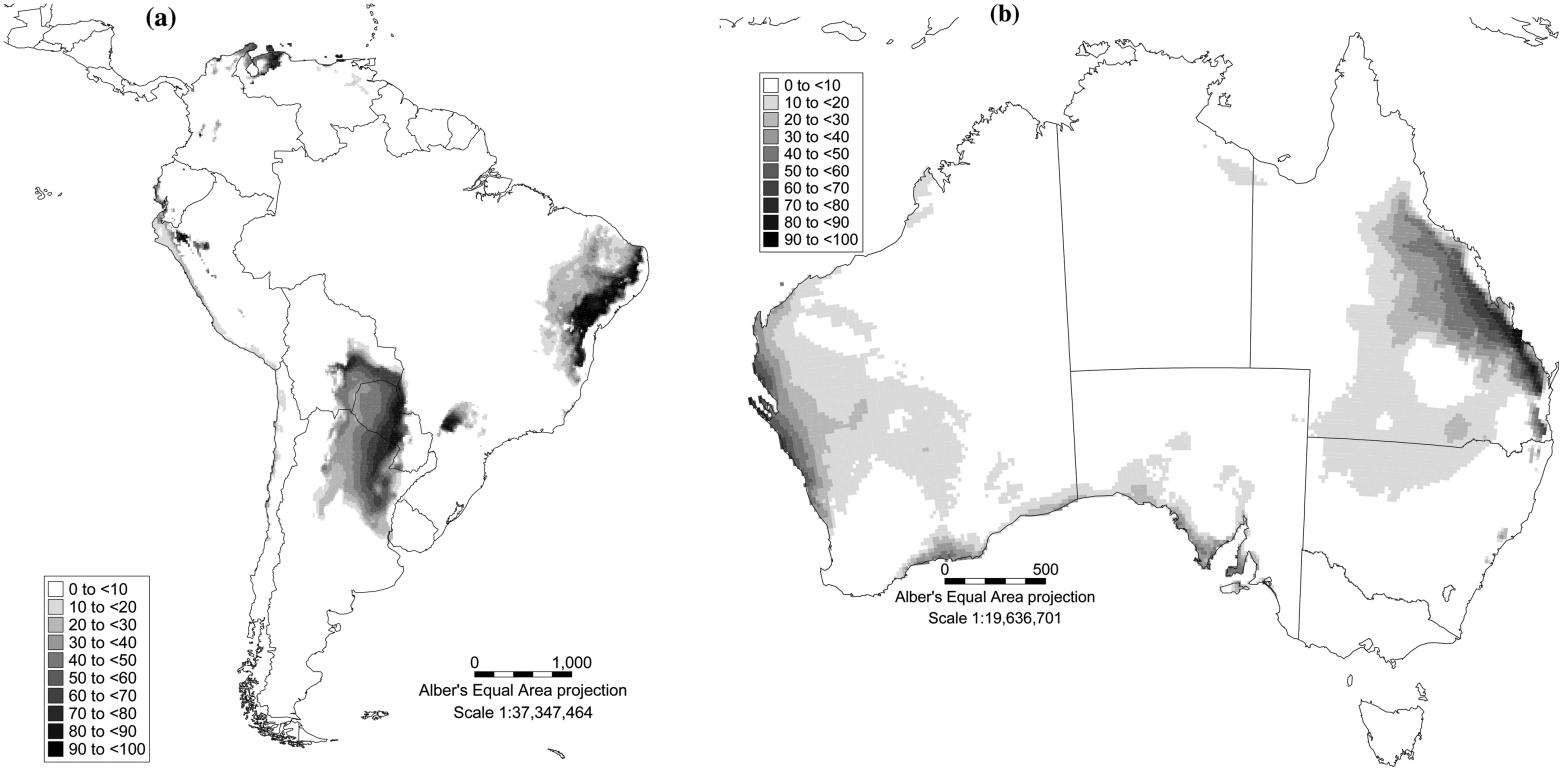
Ecoclimatic suitability (EI) for the; (a) *Harrisia martinii* in South America and; (b) *Harrisia martinii* in Australia.

Using the EIs generated by CLIMEX, the potential distributions of *Hy. pungens* and *H. martinii* in South America and Australia were estimated (Figs. 6a,b and 7a,b). The model indicates that the GI of *Hy. pungens* is higher in the Collinsville region in central Queensland than it is in the Goondiwindi region in southern Queensland for much of the year (Supplementary Fig. S1a). The GI for *Hy. pungens* is zero in the middle of winter in central Queensland, when the moisture index for the species declines to zero (Supplementary Fig. S1b). In southern Queensland, the temperature index for *Hy. pungens* (Supplementary Fig. S1c) is below that in central Queensland (Supplementary Fig. S1d) all year and the model predicts that the species suffers from significant cold stress in southern Queensland in winter, while almost no cold stress is experienced by *Hy. pungens* in central Queensland (Supplementary Fig. S1d).

The CLIMEX model indicates that although the GIs for *H. martinii* are similar in both central and southern Queensland for much of the year (Supplementary Fig. S2a), the low moisture index (Supplementary Fig. S2b), and dry stress (Supplementary Fig. S2c) experienced in central Queensland likely contribute to the much lower GI experienced in central Queensland during winter months (Supplementary Figs. S2a and b). Overall the temperature index for *H. martinii* is higher all year round in central Queensland Supplementary Fig. S2c) and although plants experience dry stress in both locations in winter (Supplementary Fig. S2d), both the intensity and the duration of cold stress are greater in southern Queensland than in central Queensland.

## Discussion

Temperature had a significant effect on the settling, development, survival, and reproduction of *Hy. pungens*. Similarly, previous studies have established that climatic factors, especially temperature, affect rates of insect development, survival, reproduction, and number of generations per year (Bale et al.1989, 2002, Coulson and Bale 1992, DeLucia et al. 2012, Pincebourde et al. 2017). In this study, laboratory results indicate that temperatures typical of cold winters and hot summers could be critical limiting factors for the performance of this mealybug in different locations in Australia. The *Hy. pungens* first instars were able to settle on host plants across a wide temperature range (15 to 40 °C). However, temperature had a significant effect on the survival and population growth of the mealybug. At 15 and 40 °C, all settled nymphs failed to develop and eventually died. This indicates the establishment of the mealybug may be reduced at prolonged periods below 15 °C and above 40 °C, likely reducing population growth. The observed mortality across all temperatures tested in this study occurred at the first instar stage. The first instar is the dispersal stage of *Hy. pungens*, and high mortality at this stage is likely to affect mealybug infestation rates of *H. martinii* in all seasons but effects are likely to be more pronounced in cold and hot seasons. Mortality at this life stage decreased by ≥80% between 20 and 35 °C following wax production (settling) by the mealybug, suggesting that in addition to protecting against predators (Kumar et al. 1997, Ammar et al. 2013), the mealybugs’ waxy coverings may also reduce the susceptibility of the mealybug to direct temperature-related impacts such as desiccation (Foldi and Pearce 1985).

Seasonal temperature changes will affect post-settling population increases, especially during low winter temperatures, as the development time of the mealybug was prolonged at 20 °C and its lower development threshold was 14.5 °C. This further emphasizes that low winter temperatures could be critical for the establishment of this mealybug in the field. Apart from low- and high-temperature impacts on survival and development rates, there were significant temperature effects on female size and reproduction. The size of an organism can positively correlate with potential fecundity (Rae and De’Ath 1991, Berger et al. 2008, Aguirre et al. 2016), and both female size and rate of reproduction of insects decrease with increasing temperature (Stelzer 2002, Fischer et al. 2003). Angilletta (2009) also suggests that ectothermic organisms in colder climates develop more slowly and mature with larger body sizes but produce fewer offspring. However, the effect of developmental temperature on an organism’s fitness may depend on other conditions such as host plant quality (Newman 1998, Weetman and Atkinson 2002). In this study, female mealybugs that developed at high temperatures were smaller and laid fewer eggs than those developing at lower temperatures. However, larger females at temperatures close to the lower development threshold may not necessarily demonstrate a corresponding increase in fecundity, as observed in females reared at 20 °C.

Insect performance, or fitness, could be determined by the integration of different insect life traits (eg, see Mathenge et al. 2009, Wang et al. 2020, 2022). Using development rates, survival, and fecundity as key traits, the integrative performance of *Hy. pungens* in this study was determined to be higher at 25 °C compared to the other temperatures tested, especially at 20 °C and 35 °C. This indicates that overall performance could be critically affected during seasons, where temperatures deviate significantly from 25 °C and could affect the ability of the mealybug to establish and develop on the target weed in locations where temperatures are above or below thermal optima for prolonged periods, thereby compromising its efficacy as a biological control agent. Other climatic factors such as variation in rainfall have been reported to affect the survival and establishment of biological control agents, with higher impacts predicted in locations with higher rainfalls (eg, see Moran and Hoffman 1987, Norris et al. 2002). It may be important to investigate the impact of varying rainfall patterns on the mealybug’s survival and distribution to understand how it contributes to the variation in mealybug performance between Australian locations.

*Hypogeococcus pungens* is reported to perform better as a biological control agent against *H. martinii* at sites in central Queensland compared to sites in southern Queensland (Tomley and McFadyen 1985). The reason for this may be partly due to the lower population growth of the mealybug in southern Queensland. The long-term mean temperatures indicate that the periods for *Hy. pungens* optimal performance at Collinsville is 1.8-fold greater than that at Goondiwindi. In the native range of *Hy. pungens* in Chaco, north-eastern Argentina, and in Collinsville, the mean monthly temperature never drops below the lower development threshold. In contrast, in Goondiwindi, the mean monthly temperature remains lower than the development threshold for 3 mo of the year, suggesting that *Hy. pungens* undergoes 3 mo of cold stress annually in this region. The CLIMEX models produced in this study also suggest that the mealybug will perform better in Collinsville than in Goondiwindi. In Collinsville, plant growth is limited by the interaction between moisture stress, cold stress, and dry stress, while the mealybug only suffers moisture stress. However, the impact of ambient moisture on the mealybug could be minimal as cacti are known to retain water. The model identifies cold stress as the main limiting factor affecting the mealybug growth in Goondiwindi, and while *H. martinii* also suffers cold stress at this location, it is less intense and has minimal impact on its growth. Hassan et al. (2021) reported that cold stress affects plant growth, but most cactus species are known to be cold-tolerant (Nobel 1982). This may be the reason cold stress has more impact on the mealybugs’ growth than it does on the plants in Goondiwindi.

Cold stress affects insect development, fecundity, and survival (Rinehart et al. 2000, Zizzari and Ellers 2011, Seehausen et al. 2017) and it can also affect other life processes, such as flight, muscular, and neural functions (Klose et al. 2004, Chown and Terblanche 2006). The impact of cold temperatures on insects depends on the intensity of the low temperature and length of exposure (Robinet and Roques 2010). In winter, Goondiwindi temperature sometimes falls well below zero at night, which further exposes the mealybug to extreme cold temperature impacts. Future investigation into the mealybug’s critical thermal minima as well as the effects of prolonged cold stress and freezing temperature on *Hy. pungens* could provide more insight on the impact of cold stress accumulation on mealybug performance. This study has provided a good understanding of the relationship between temperature and life traits of *Hy. pungens*, which is crucial information in the ongoing biological control programme using this agent.

In summary, this study suggests that seasonal temperature variation is an important factor that affects the overall fitness and performance of *Hy. pungens* differently at different locations in Australia. Although the establishment of the mealybug is possible in all locations where *H. martinii* occurs in Queensland, its performance will vary between locations due to variations in development and population growth rates caused by local climatic conditions. The interaction between slow or no development and low reproduction rates at low and high temperatures will have a critical impact on *Hy. pungens* performance in Queensland locations such as Goondiwindi which experience very low winter and high summer temperatures. A study of this nature is important as it could help in predicting the distribution of insects in different temperature ranges, and provide a better understanding of insects and their interactions with their host plants and natural enemies (Robertson et al. 2003, Zalucki and Klinken 2006, Olfert et al. 2016, Venter et al. 2022).

## Supplementary material

Supplementary material is available at *Environmental Entomology* online.

nvaf026_suppl_Supplementary_Figure_S1

nvaf026_suppl_Supplementary_Figure_S2
