# Supplementary figures and images for: Thermal biology of Hypogeococcus pungens (Hemiptera: Pseudococcidae) explains its variable performance as a classical biological control agent for Harrisia martinii (Cactaceae) in Australia
Source: Environ Entomol. 2025 Apr 12;54(3):454–66. doi: 10.1093/ee/nvaf026 (PMC12199162; doi:10.1093/ee/nvaf026)

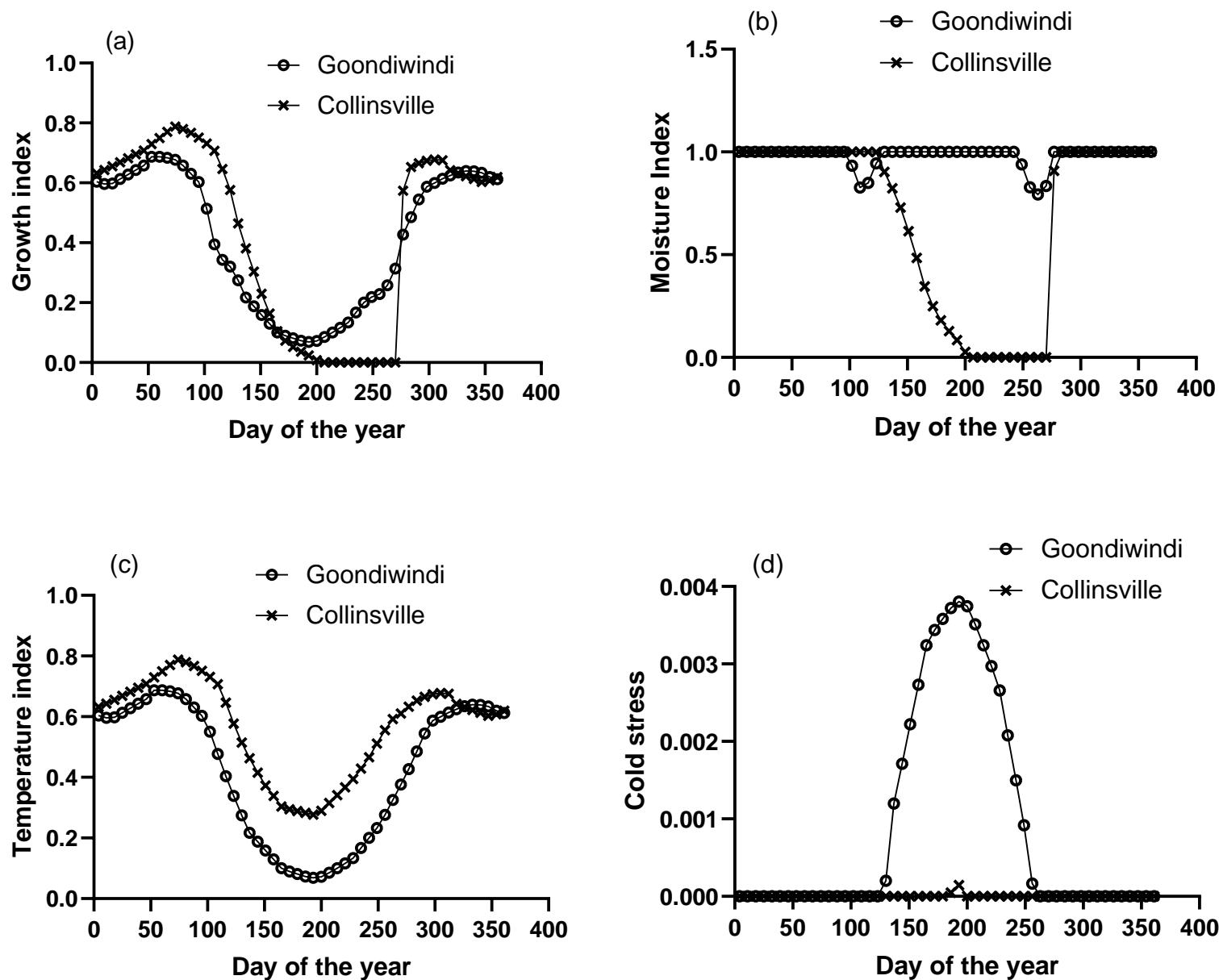

Supplementary Fig. 1.

Supplement: nvaf026_suppl_Supplementary_Figure_S1 [file nvaf026_suppl_supplementary_figure_s1.pdf]

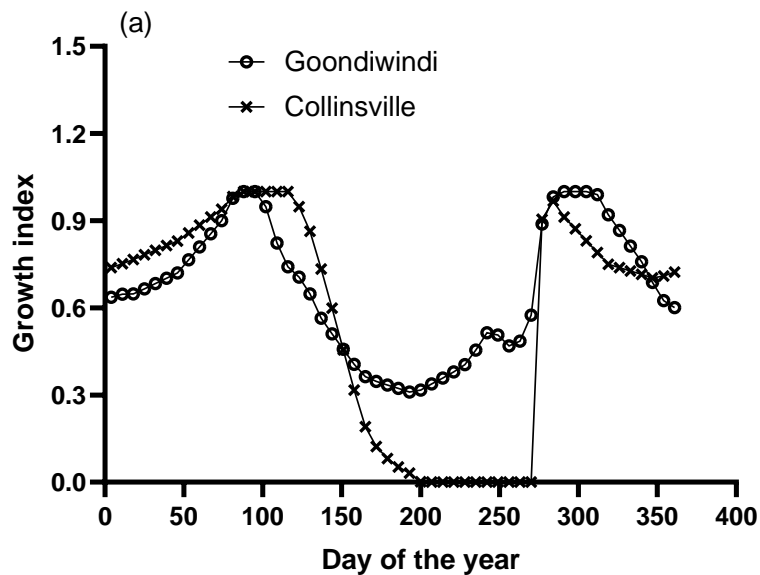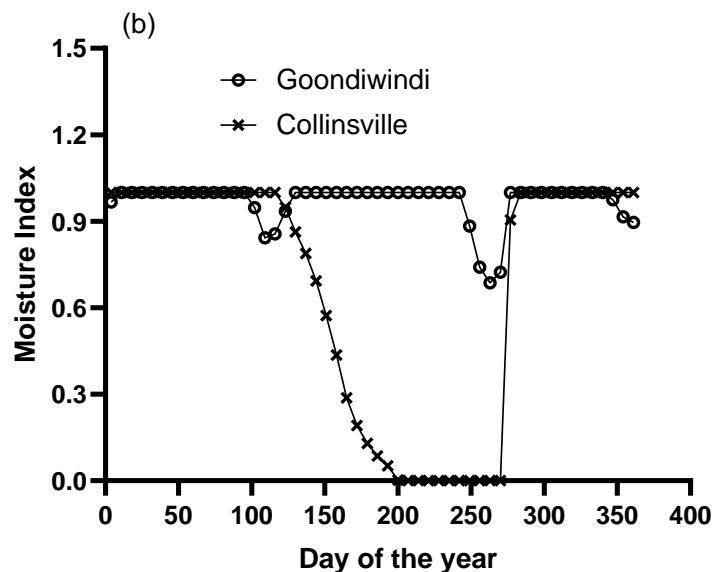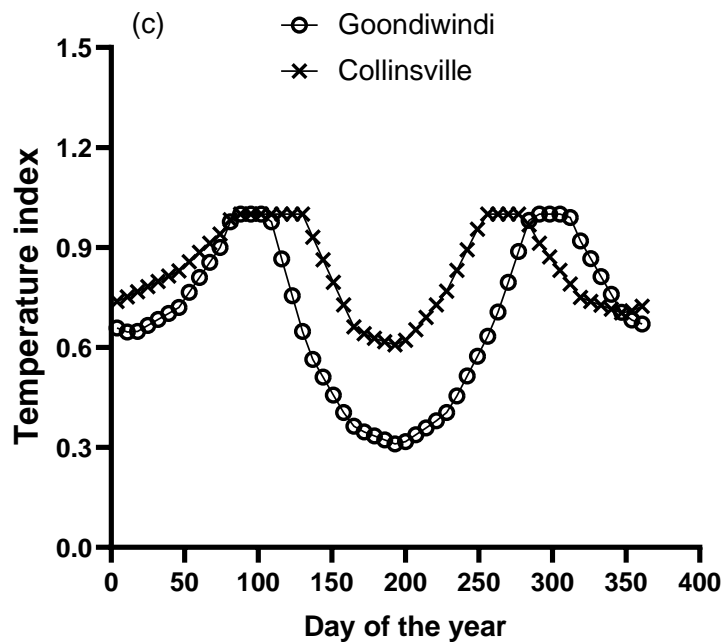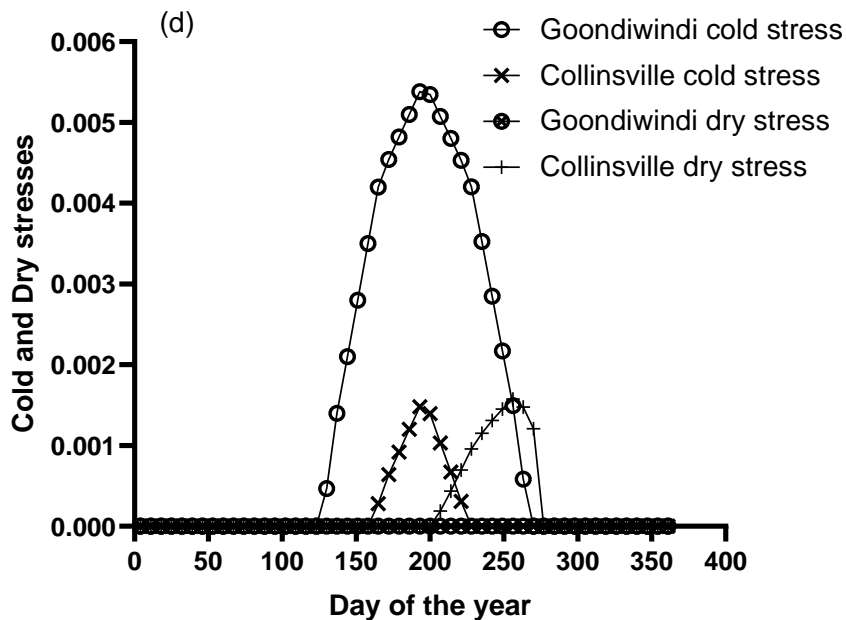

Supplementary Fig. 2

Supplement: nvaf026_suppl_Supplementary_Figure_S2 [file nvaf026_suppl_supplementary_figure_s2.pdf]
